# Associations of Serum and Urinary Magnesium with the Pre-Diabetes, Diabetes and Diabetic Complications in the Chinese Northeast Population

**DOI:** 10.1371/journal.pone.0056750

**Published:** 2013-02-13

**Authors:** Jiancheng Xu, Wei Xu, Hanxin Yao, Weixia Sun, Qi Zhou, Lu Cai

**Affiliations:** 1 The Department of Clinical Laboratory at the First Hospital of Jilin University, Changchun, People's Republic of China; 2 The Department of Pediatrics at the First Hospital of Jilin University, Changchun, People's Republic of China; 3 The Kosair Children Hospital Research Institute at the Department of Pediatrics of University of Louisville, Louisville, Kentucky, United States of America; University of Kentucky, United States of America

## Abstract

The effect of magnesium (Mg) deficiency on the prevalence of diabetes and diabetic complications has received a great attention. The present study investigated the association of Mg level in the serum or urine of the patients, lived in the Northeast areas of China, with either pre-diabetes or diabetes with and without complications. From January 2010 to October 2011, patients with type 1 diabetes (T1D, *n* = 25), type 2 diabetes (T2D, *n* = 137), impaired fasting glucose (IFG, *n* = 12) or impaired glucose tolerance (IGT, *n* = 15), and age/gender matched control (n = 50) were enrolled in the First Hospital of Jilin University. In T2D group, there were 24, 34, and 50 patients with nephropathy, retinopathy or peripheral neuropathy. Serum Mg levels in the patients with IGT, IFG, T2D, and T1D were significantly lower than that of control. The urinary Mg levels were significantly increased only in T2D and T1D patients compared to control. There was no difference for these two changes among T2D with and without complications; In addition, there was a significantly positive correlation of serum Mg levels with serum Ca levels only in T2D patients, and also a significantly positive correlation of urinary Mg levels with urinary Ca levels in control, IGT patients, and T2D patients. Simvastatin treatment in T2D patients selectively reduced serum Ca levels and urinary Mg levels. These results suggest that the potential impact of Mg deficiency on metabolic syndrome, diabetes and diabetic complications needs to be received special attention.

## Introduction

Diabetes is a growing public health burden across the world, particularly in the developing countries [1]–[3]. The prevalence of diabetes in China has reached epidemic proportions, affecting about 92.4 million people aged ≥20 years (9.7% of the adult population) [3]. The threatening effect of diabetes for these patients is its complications, including cardiovascular and neuronal disorders [3], [4]. Diet is widely believed to play an important role in the development of type 2 diabetes (T2D) and the associated complications [3], [4].

Homeostasis of the trace elements such as zinc, copper, iron, and magnesium (Mg) has been found to play an important role in the pathogenesis of diabetes and diabetic complications [5]–[9]. Mg, one of the important components of many foods such as grains, nuts, and green leafy vegetables, is the fourth most abundant cation in our body and plays a key role in many fundamental biological processes, including energy metabolism. Mg has received considerable attention for its potential in improving insulin sensitivity and preventing diabetes and its cardiovascular complications [10]–[13]. However, results are inconsistent among the studies [14], [15]. By following-up for 6 years, for instance, Atherosclerosis Risk in Communities Study group examined the risk for T2D in over 12,000 middle-aged adults without diabetes at baseline examination, but they did not find any statistical association between dietary Mg intake and incidence of T2D in either black or white research subjects [15]. In addition, observations in Caucasian diabetics have linked hypomagnesaemia as being an additional risk factor for the development of diabetic retinopathy (DR), but this correlation was not observed in black African diabetics [14]. Therefore, inconsistent findings for the correlation of serum Mg with the risk of diabetes and diabetic complications is not only attributed to the difference in population [14], [15], but also attributed to difference in the measurements [16].

There was no much information for the effect of serum Mg levels on the prevalence of diabetes and diabetic complications, based on Chinese population [17]. Therefore, we have examined the Mg levels in the serum and urine of Northeast Chinese population at different categories of insulin sensitivity (metabolic syndrome) and diabetes with and without diabetic complications.

## Research Design and Methods

### Ethics Statement

This study was approved by the institutional ethics committee of the First Hospital of Jilin University and written informed consent was obtained from all subjects before their enrollment into the study. For the patients who were younger than 18 year old, the informed consents were obtained from their parent by written.

### Patients and their general information

From January 2010 to October 2011, there were 189 patients and 50 healthy control enrolled (30 males and 20 females, at the age range of 20–59 with a median age of 50 years old). These patients were diagnosed as impaired fasting glucose (IFG, *n* = 12, 8 males and 4 females, age range of 31–53), IGT (*n* = 15, 9 males and 6 females with an age range of 40–56), type 1 diabetes (T1D, *n* = 25, 8 males and 17 females with an age range of 9–33 years at median age of 25 years), and T2D (*n* = 137, 85 males and 52 females, age range of 42–62 with a median age of 56 years old). Among the patients with T2D, patients with nephropathy (DN, *n* = 24, 19 males and 5 females, with a median age of 60 from 28 to 84 years old), and patients with DR (*n* = 34, 15 males and 19 females, ages from 29 to 74 with a median age at 60 years old), and peripheral neuropathy (DPN, *n* = 50, 29 males and 21 females, ages from 27 to 79 with a median age of 56 years old).

Demographic data for these patients, including age, sex, BMI, presence or absence of diabetes, hypertension, dyslipidemia, and medication (simvastatin), were obtained from the patients' medical records. Body mass index (BMI) was calculated as body weight (kg) divided by height (m) squared. Hypertension was defined as systolic blood pressure ≥140 mm Hg and/or diastolic blood pressure ≥90 mm Hg as measured by automatic devices with the patient in a sitting position and/or a previous diagnosis of hypertension.

**Impaired fasting glucose** (IFG) was defined as a fasting glucose concentration 6.1–6.9 mmol/L and nonfasting glucose concentration <7.8 mmol/L.

**Impaired glucose tolerance** (IGT) was defined as a fasting glucose <7.0 mmol/L, but nonfasting glucose level in 7.8–11.0 mmol/L.

**Diabetes** was defined as a fasting glucose concentration ≥7.0 mmol/L, non-fasting glucose concentration ≥11.1 mmol/L, hemoglobin A1c (HbA1c) value ≥6.5%, and/or a previous diagnosis of diabetes.

Dyslipidemia was defined as a triglyceride level ≥1.7 mmol/L, total cholesterol ≥5.18 mmol/L, low density lipoprotein (LDL) ≥3.37 mmol/L, and/or previous diagnosis of dyslipidemia. Estimating glomerular filtration rate (eGFR) was used as measure of kidney function.

### Other measurements

Laboratory data including glucose, HbA1c, red blood cell (RBC), hemoglobin, blood urea nitrogen (BUN), creatinine, total cholesterol (CHO), triglyceride (TG), high-density lipoprotein (HLDL), low-density lipoprotein cholesterol (LDL) levels were measured using standard methodology from the first blood samples, which were obtained from most participants soon after admission. Mg and Ca were assessed using inductively coupled plasma (ICP) on Shimadzu multitype ICP emission spectrometer. A 24-h urine sample was obtained after admission to measure eGFR.

Blood samples from subjects were taken after overnight fasting into commercial tubes for analysis of laboratory parameters and into special metal-free tubes for analysis of Ca and Mg using the standard venipuncture technique. After blood centrifugation, serum was aliquoted into metal-free Eppendorf test tubes, shock frozen, and stored at −80°C until further analysis.

The eGFR was calculated based on the Cockroft-Gault equation for Chinese individual: Creatinine Clearance (Ccr) = [(140-age)×body weight]/(serum creatinine×72)×0.85 (if female) [18].

Participants were categorized by serum Mg level with a cutoff value of 18 mg/L (Low-Mg group, serum Mg level ≤18 mg/L; High-Mg group, serum Mg level>18 mg/L). This cutoff value was chosen based on the previously published normal lower limit [19].

### Comparison of serum Mg levels in the T2D patients before and after 1-month treatment with simvastatin

Twenty four patients with T2D who were not originally with any lipid-lowering drug were recruited. Inclusion criteria were CHO above 6.22 mmol/L and LDL above 4.14 mmol/L. Exclusion criteria were therapy with lipid-lowering drugs including fish oil, probucol, vitamin E, steroid hormones, immunosuppressants, aluminum-containing antacids and erythromycin, ketoconazole, analogues or p-aminoacetic acid. Patients were assigned to treatment for 1 month with 10 mg/day of simvastatin as clinically indicated. Blood samples were taken from the fasted patients at the beginning and the end of the 1-month therapy.

### Statistical analysis

Continuous variables were expressed as median (interquartile range) and categorical variables as number (percent). Mann-Whitney U-test was used for comparisons between groups, depending on the distribution. Differences in frequency of categorical variables were assessed by the chi-square test or Fisher's exact test as appropriate. Spearman rank correlation analysis was used to evaluate the correlations between serum Mg or urinary Mg level as a continuous variable and laboratory parameters. Baseline characteristics were adjusted for age, sex, BMI, hypertension and dyslipidemia by analysis of covariance using general linear models. All reported *P* values were two-sided, and values of *P*<0.05 were considered statistically significant. Statistical analyses were performed using SPSS 17.0.

## Results

### Study population and baseline characteristics

**Figure 1:**
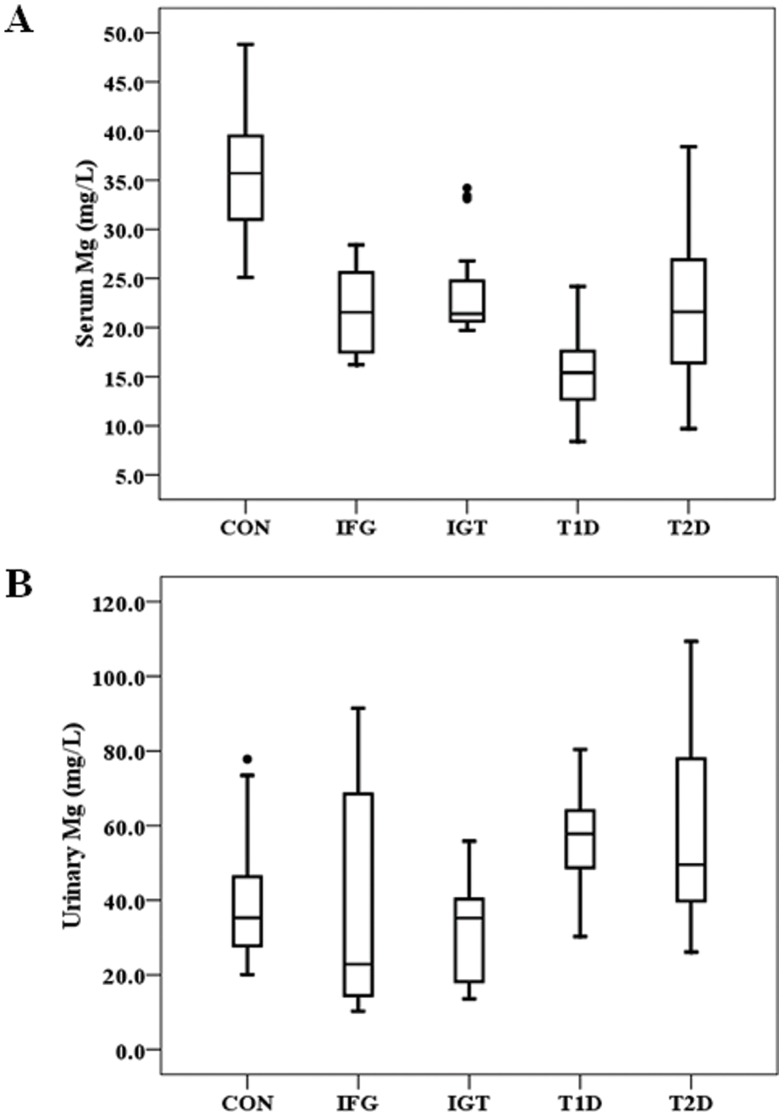
Levels of Mg in healthy control, IFG, IGT, T1D and T2D groups. Boxplots display the extremes, the upper and lower quartiles, and the median of the maximum difference in healthy control, IFG, IGT, T1D and T2D groups. The median for each dataset is indicated by the centerline, and the first and third quartiles are represented by the edges of the area, which is known as the inter-quartile range (IQR). The extreme values (within 1.5 times of the IQR from the upper or lower quartile) are represented by the ends of the lines extending from the IQR. Points at a greater distance from the median than 1.5 times of the IQR are plotted individually as dots. *P* value of serum Mg levels was <0.0001 between healthy control group and IFG, IGT, T1D and T2D groups (Fig. 1A). *P* value of urinary Mg levels was <0.0001 between healthy control group and T1D and T2D groups (Fig. 1B).

Analysis of the serum level of Mg showed that compared to control, the serum Mg level was low in IFG, IGT, T2D, and T1D (Fig. 1A). The urinary Mg level in the patients with either T1D or T2D was significantly high compared to control while it in the patients with IFG or ITG was not significantly changed (Fig. 1B).

**Figure 2:**
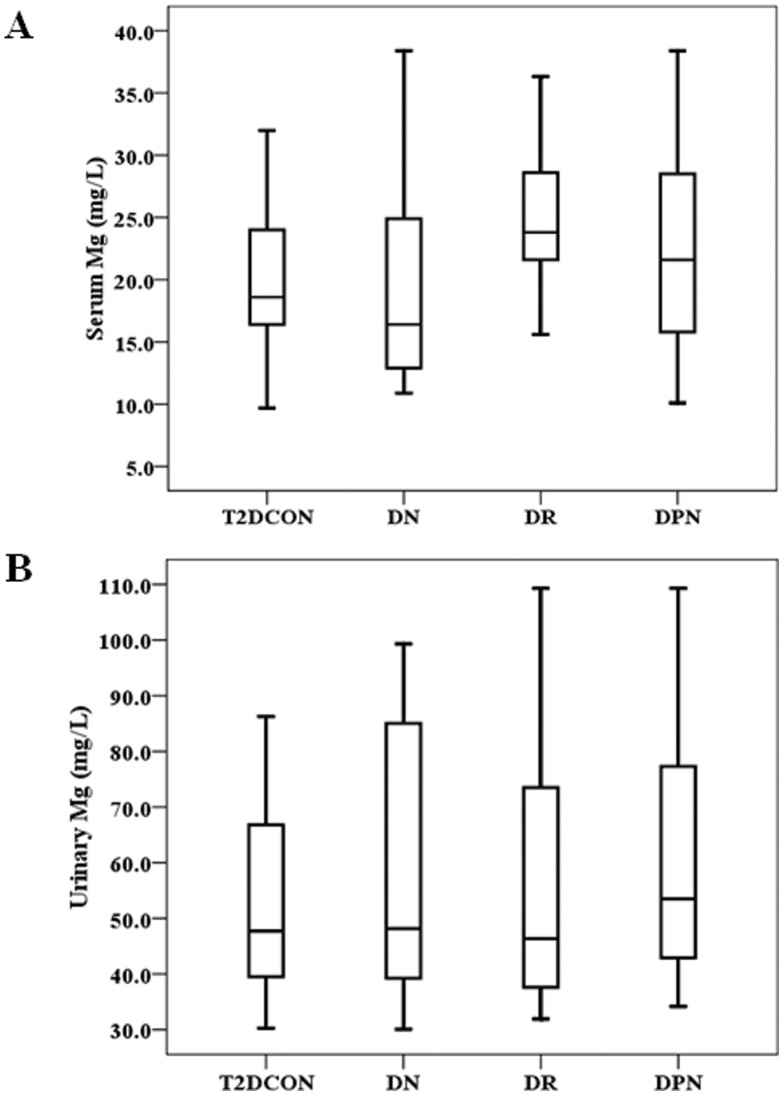
Levels of Mg in T2D without complications, DN, DR, and DPN groups. *P* value of serum Mg levels was <0.01 between T2D without complications (T2DCON) and DR group (Fig. 2A). *P* value of urinary Mg levels was >0.05 between T2DCON and DN, DR or DPN groups (Fig. 2B).

Among the patients with T2D, we further compared the serum and urinary levels of Mg between those who do not have diagnosed complications, including DN, DR or DPN, and those who have one of the these complications. Fig. 2A showed that only serum Mg level was higher in DR T2D patients than that in uncomplicated T2D patients. There was no significant difference for the urinary Mg level between uncomplicated T2D patients and T2D with DN, DR or DPN (Fig. 2B).

### Analysis of serum Mg as low and high levels within groups

Since T2D patients have shown a clear decrease in the serum Mg level (Fig. 1A), we further divided these patients into Low-Mg (serum Mg level ≤1.8 mg/L) and High-Mg (serum Mg level >1.8 mg/L) groups with the previously published normal lower limit chosen as the cutoff point. Table 1 presents the summary of basal characteristics stratified by Mg groups in the control (serum Mg levels were all >1.8 mg/L) and T2D patients. Among the variables measured the age seems to affect the serum Mg level, showing a positive association of the age with serum Mg level. In Low-Mg group, male patients were predominant (77.1%) compared to that in High-Mg group (53.9%). In addition, significantly positive associations of serum Mg level with serum Ca level or urinary Mg were established in T2D patients. BUN and creatinine (Cre) levels were significantly increased in T2D patients with Low-Mg compared to those with High-Mg, while RBC was slightly increased in T2D with Low-Mg compared to those with High-Mg.

**Table 1:** Baseline characteristics stratified by serum Mg levels in T2D subjects.

	T2D (*n* = 137)			CON (*n* = 50)
	Low-Mg (*n* = 48)	High-Mg (*n* = 89)	*P*	High-Mg (*n* = 50)
Age (years)	52 (44–60)	58 (47–65)	0.038*	55 (45–63)
Sex, male (%)	37 (77.1)	48 (53.9)	0.008*	31 (62.0)
BMI (kg/m^2^)	25.6 (23.0–27.7)	25.2 (23.3–27.5)	0.799	21.6 (19.5–22.9)
Glu (mmol/L)	8.5 (7.3–11.7)	8.8 (7.7–11.2)	0.434	4.8 (4.1–5.6)
HbA1c (%)	8.3 (7.0–8.9)	8.3 (7.3–9.1)	0.472	5.1 (4.2–6.0)
RBC (×10^12^)	4.9 (4.6–5.1)	4.7 (4.2–5.0)	0.026*	4.8 (4.3–5.0)
Hb (g/L)	146.0 (136.3–156.3)	139.0 (127.0–153.0)	0.235	133.2 (128.1–153.7)
BUN (mmol/L)	6.5 (5.1–12.1)	5.9 (5.2–7.9)	0.031*	5.5 (4.2–6.4)
Cre (µmol/L)	81.1 (65.5–238.8)	76.9 (63.6–112.8)	0.005*	73.9 (63.1–80.9)
eGFR (ml/min)	94.0 (37.9–117.6)	85.7 (57.6–110.9)	0.656	24.6 (22.7–26.4)
Hypertension (%)	18 (37.5)	40 (44.9)	0.402	0
Dyslipidemia (%)	30 (62.5)	47 (52.8)	0.277	0
Ca (mg/L)	63.0 (53.6–72.6)	107.6 (92.5–138.9)	0.000*	165.0 (145.8–177.2)
UCa (mg/L)	94.2 (43.4–151.6)	79.2 (38.2–136.6)	0.391	63.3 (31.0–94.1)
UMg (mg/L)	54.8 (40.6–79.6)	47.7 (39.4–73.5)	0.048*	34.4 (15.1–47.3)
CHO (mmol/L)	5.0 (4.4–5.9)	5.0 (4.4–6.3)	0.234	4.5 (3.2–5.1)
TG (mmol/L)	1.6 (1.0–2.8)	1.5 (1.1–2.7)	0.578	1.2 (1.0–1.4)
HDL (mmol/L)	1.1 (0.9–1.5)	1.2 (1.1–1.5)	0.514	1.2(1.1–1.3)
LDL (mmol/L)	3.2 (2.6–3.8)	3.4 (2.7–3.9)	0.426	2.4 (2.0–2.9)

Data are presented as number (%) or median (interquartile range). Baseline characteristics were adjusted for age, sex, BMI, hypertension and dyslipidemia by analysis of covariance using general linear models.

* , *P*<0.05 vs High-Mg group. BMI: body mass index, Glu: serum glucose, HbA1c: glycated hemoglobin, RBC: red blood cell, Hb: hemoglobin, BUN: blood urea nitrogen, Cre: serum creatinine, eGFR: estimating GFR, Ca: serum calcium, UCa: urinary calcium, Mg: serum magnesium, Umg: urinary magnesium, CHO: total cholesterol, TG: triglyceride, HDL: high-density lipoprotein cholesterol, LDL: low-density lipoprotein cholesterol.

We further divided the T2D patients into those with and without DN or DPN for analyzing their serum Mg status (Table 2). Clearly serum levels of Mg were positively correlated with serum Ca levels in DN and DNP groups, but not in T2D CON group. The positive association of the age with serum Mg level, mentioned above, was only seen in the uncomplicated T2D group. The gender different distribution in two groups was only observed in T2D with DPN, i.e.: 77.8% of patients with DPN are male in Low-Mg group. Low-Mg group has a risk for the increased RBC and Hb levels only in DNP; Also only in DPN, serum Mg levels were negatively correlated with urinary Mg. There was no association of serum Mg with BUN or eGFR in any subgroup, which was seen in total T2D group (Table 1).

**Table 2:** Baseline characteristics stratified by Mg levels in T2D subjects.

	T2D Con (*n* = 29)			DN (*n* = 24)			DPN (*n* = 50)		
	Low-Mg (*n* = 14)	High-Mg (*n* = 15)	*P*	Low-Mg (*n* = 13)	High-Mg (*n* = 11)	*P*	Low-Mg (*n* = 18)	High-Mg (*n* = 32)	*P*
Age (years)	40 (28–50)	52 (42–61)	0.007*	60 (50–73)	60 (45–65)	0.663	53 (46–60)	57 (49–65)	0.460
Sex, male (%)	10 (71.4)	12(80.0)	0.682	11 (84.6)	8 (72.7)	0.484	14 (77.8)	15 (46.9)	0.035*
BMI (kg/m^2^)	25.0 (22.8–27.8)	25.7 (23.9–27.0)	0.556	26.5 (25.1–29.7)	28.7 (24.8–30.4)	0.685	25.7 (23.3–27.6)	25.0 (22.9–26.1)	0.363
Glu (mmol/L)	10.8 (8.7–13.4)	9.2 (7.8–12.9)	0.839	7.7 (7.0–9.8)	8.4 (7.2–9.0)	0.802	7.8 (7.2–10.4)	8.8 (7.6–11.5)	0.162
HbA1c (%)	8.7 (8.3–9.5)	8.0(7.4–9.3)	0.883	8.4 (7.4–9.8)	8.3 (7.4–9.8)	0.262	7.3 (6.5–8.5)	8.1 (6.7–8.9)	0.271
RBC (×10^12^)	5.2 (4.6–5.4)	5.1 (4.6–5.4)	0.066	4.9 (4.3–5.1)	4.7 (3.4–5.4)	0.120	4.9 (4.7–5.0)	4.5 (4.1–4.7)	0.015*
Hb (g/L)	152.0 (131.0–157.0)	158.0 (142.0–164.0)	0.073	145.0 (133.5–156.0)	137.0 (129.0–160.0)	0.137	146.0 (137.0–155.0)	135.0 (125.0–148.0)	0.019*
BUN (mmol/L)	5.5 (4.7–6.6)	5.6 (4.5–6.3)	0.631	18.7 (14.6–21.0)	14.2 (11.9–17.5)	0.120	5.8 (4.2–6.5)	6.1 (4.2–7.4)	0.107
Cre (µmol/L)	68.1 (53.3–78.1)	75.6 (60.8–87.9)	0.362	341.5 (269.5–418.9)	234.7 (185.4–288.5)	0.116	75.3 (64.8–84.6)	66.4 (60.7–89.4)	0.569
eGFR (ml/min)	114.7(88.3–190.6)	94.5(84.5–115.3)	0.420	17.9 (14.0–28.7)	25.7 (20.1–39.3)	0.233	99.2 (85.6–115.9)	93.2 (66.3–111.6)	0.915
Hypertension (%)	0	0	-	10 (76.9)	11 (100.0)	0.095	7 (38.9)	13 (40.6)	0.905
Dyslipidemia (%)	9 (64.3)	11 (73.3)	0.605	8 (61.5)	6 (54.5)	0.735	12 (66.7)	15 (46.9)	0.182
Ca (mg/L)	91.2 (63.2–100.8)	109.6 (98.5–115.7)	0.153	62.7 (49.4–66.8)	138.9 (91.3–153.3)	0.002*	56.4 (53.2–66.0)	103.7 (78.0–139.5)	0.001*
UCa (mg/L)	100.8 (72.6–173.6)	79.2 (50.2–138.6)	0.005*	29.4 (10.7–149.6)	73.5 (12.7–216.9)	0.622	99.5 (71.4–154.7)	87.1 (58.7–116.4)	0.076
UMg (mg/L)	55.3 (39.1–80.8)	47.3 (42.2–53.8)	0.030*	42.9 (38.7–66.1)	57.6 (43.7–93.4)	0.643	64.9 (49.8–90.9)	49.6 (41.5–75.5)	0.041*
CHO (mmol/L)	4.9 (4.1–5.3)	5.2 (4.6–5.6)	0.467	5.0 (4.5–6.2)	6.1 (5.6–6.8)	0.212	5.0 (4.2–6.0)	4.8 (4.3–6.0)	0.678
TG (mmol/L)	1.8 (1.1–2.4)	1.7 (1.1–3.3)	0.665	1.8 (1.0–3.2)	2.0 (1.9–4.2)	0.157	1.5 (1.1–3.0)	1.3 (1.0–2.4)	0.704
HDL (mmol/L)	1.0 (0.9–1.6)	1.3 (0.8–1.6)	0.330	1.2 (0.9–1.6)	1.1 (1.1–1.5)	0.523	1.1 (0.9–1.3)	1.2 (1.1–1.4)	0.504
LDL (mmol/L)	3.0 (2.1–3.5)	3.5 (2.7–3.9)	0.617	3.3 (2.8–4.0)	3.8 (3.2–4.2)	0.645	3.1 (2.6–3.7)	3.3 (2.6–4.0)	0.520

Data presentation and abbreviation's spell are same as the description for Table 1.

* , *P*<0.05 vs High-Mg group.

### Correlating analysis of serum or urinary Mg with Ca and variables related to renal function

**Figure 3:**
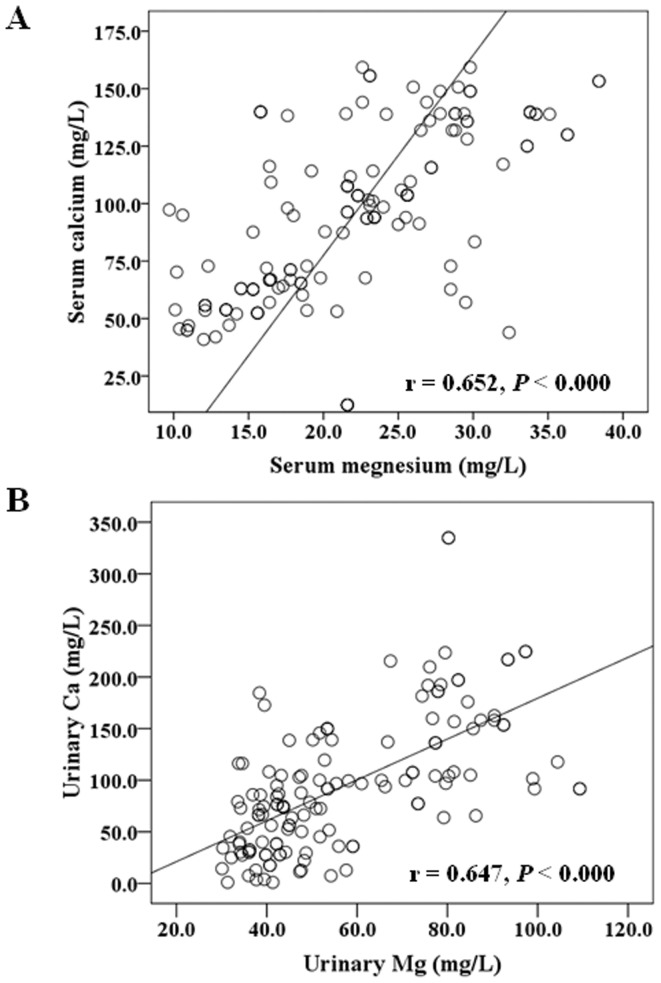
Correlation between Mg with Ca in T2D patients. Correlations between serum Mg and serum Ca in T2D patients (r = 0.652, *P*<0.000, Fig. 3A). Correlations between urinary Mg and urinary Ca in T2D patients (r = 0.647, *P*<0.000, Fig. 3B).

Since the above analyses (based on two groups of serum Mg levels) revealed certain associations of serum Mg levels with serum Ca or urinary Mg levels, we further individually analyze the correlation of the Mg level with Ca either in serum or urine, and also the possible correlation with renal function within the group of control, IFG, IGT, T1D and T2D (Table 3). It is clearly shown that in all groups, neither serum Mg levels nor urinary Mg levels have any correlation with renal function measurements, including BUN, Cre, and eGFR. There was no any correlation of serum Mg or urinary Mg level with serum Ca or urinary Ca level, except for a positive correlation of serum Mg with serum Ca in uncomplicated T2D group (*P*<0.000), for which the correlation curve is presented in Fig. 3A. In addition, urinary Mg levels were found to be positively correlated with urinary Ca in control, IGT, or T2D group. The correlation curve for T2D group is presented in Fig. 3B.

**Table 3:** Associations between serum Mg or urine Mg level as a continuous variable and laboratory parameters.

	CON (*n* = 50)				IFG (*n* = 12)				IGT (*n* = 15)				T1D (*n* = 25)				T2D (*n* = 137)			
	Mg		U Mg		Mg		U Mg		Mg		U Mg		Mg		U Mg		Mg		U Mg	
	R	*P*	r	*P*	r	*P*	r	*P*	r	*P*	r	*P*	r	*P*	r	*P*	r	*P*	r	*P*
UMg	−0.203	0.157	-	-	−0.420	0.174	-	-	0.025	0.930	-	-	0.312	0.130	-	-	−0.064	0.458	-	-
Ca	0.192	0.183	−0.069	0.635	0.086	0.791	0.282	0.374	−0.129	0.648	−0.370	0.175	0.217	0.298	0.195	0.349	0.652	0.000*	0.056	0.517
UCa	−0.086	0.553	0.345	0.014*	−0.223	0.486	−0.239	0.454	0.254	0.362	0.721	0.002*	−0.165	0.429	0.304	0.140	−0.056	0.515	0.647	0.000*
BUN	−0.067	0.646	−0.202	0.158	0.301	0.341	−0.277	0.384	0.133	0.638	0.251	0.367	0.265	0.201	0.189	0.366	−0.064	0.455	0.101	0.240
Cre	0.001	0.993	0.081	0.577	−0.368	0.240	0.354	0.259	0.241	0.386	0.395	0.145	0.300	0.146	0.180	0.388	−0.062	0.470	−0.069	0.421
eGFR	−0.022	0.880	0.011	0.938	0.165	0.609	−0.280	0.378	0.086	0.761	−0.218	0.435	−0.238	0.252	−0.296	0.151	0.004	0.962	0.031	0.716

Data presentation and abbreviation's spell are same as the description for Table 1.

* , *P*<0.05 for the association.

We further divided T2D patients into those uncomplicated (T2D Con) and with DN, DR or DPN to correlatively analyze these measurements (Table 4). Following characteristics were found: (1) Serum Mg level was negatively correlated with urinary Mg level only in DPN group (*P*<0.05); (2) Serum Mg level was positively correlated with serum Ca in all groups (*P*<0.01); (3) Urinary Mg level was positively correlated with urinary Ca in all groups (*P*<0.01); (4) Urinary Mg level was positively correlated with BUN level only in uncomplicated T2D (*P*<0.01); (5) Only in DN group, serum Mg level was negatively correlated with creatinine (*P*<0.01) while urinary Mg level may be positively correlation with eGFR (*P* = 0.05).

**Table 4:** Associations between serum Mg or urine Mg level as a continuous variable and laboratory parameters.

	T2D Con (*n* = 29)				DN (*n* = 24)				DR (*n* = 34)				DPN (*n* = 50)			
	Mg		U Mg		Mg		U Mg		Mg		U Mg		Mg		U Mg	
	r	*P*	r	*P*	r	*P*	r	*P*	r	*P*	r	*P*	r	*P*	r	*P*
UMg	−0.128	0.509	-	-	0.169	0.430	-	-	0.137	0.438	-	-	−0.302	0.033*	-	-
Ca	0.595	0.001*	−0.165	0.394	0.877	0.000*	0.392	0.058	0.462	0.006*	0.291	0.096	0.676	0.000*	−0.151	0.294
UCa	−0.328	0.083	0.493	0.007*	0.073	0.734	0.754	0.000*	0.196	0.267	0.536	0.001*	−0.149	0.302	0.804	0.000*
BUN	−0.137	0.478	0.574	0.001*	−0.229	0.123	−0.084	0.568	−0.154	0.385	0.100	0.576	0.241	0.091	−0.033	0.818
Cre	−0.288	0.130	−0.133	0.493	−0.393	0.008*	−0.022	0.882	−0.071	0.691	−0.044	0.805	−0.062	0.667	−0.157	0.276
eGFR	−0.273	0.152	−0.083	0.670	0.272	0.066	0.288	0.050*	0.125	0.481	0.067	0.706	−0.125	0.387	0.079	0.584

Data presentation and abbreviation's spell are same as the description for Table 1.

* , *P*<0.05 for the association.

### Effect of simvastatin treatment on Mg and Ca levels and other variables in T2D patients

Since T2D patients were often treated with statins to lower their lipid profiles, we examined the effect of 1-month simvastatin treatment on Mg and Ca levels in T2D patients (Table 5). Treatment with simvastatin for 1 month significantly reduced CHO and LDL levels as expected, and also significantly reduced serum Ca level (*P*<0.05) and urinary Mg level (*P*<0.01).

**Table 5:** Serum parameters in T2D patients treated with simvastatin.

	Simvastatin (*n* = 24)		
	Pretreatment	posttreatment	*P*
Glu (mmol/L)	7.7 (7.3–8.4)	7.7 (6.9–8.3)	0.288
HbA1c (%)	7.7 (6.4–8.8)	7.6 (6.5–8.0)	0.278
CHO (mmol/L)	5.7 (4.9–6.5)	4.4 (3.6–5.1)	0.001*
TG (mmol/L)	1.3 (0.9–2.3)	1.5 (1.1–2.2)	0.421
HDL (mmol/L)	1.2 (1.1–1.7)	1.3 (1.0–1.7)	0.869
LDL (mmol/L)	3.8 (3.4–4.4)	2.7 (1.8–2.8)	0.000*
Apo-a (mmol/L)	0.09 (0.06–0.13)	0.08 (0.04–0.12)	0.549
Ca (mg/L)	69.1 (58.1–121.3)	57.5 (45.9–81.9)	0.012*
UCa (mg/L)	72.4 (48.7–115.4)	55.8 (45.0–125.6)	0.621
Mg (mg/L)	18.6 (14.6–28.1)	18.3 (14.5–28.1)	0.635
UMg (mg/L)	53.2 (42.8–80.5)	39.3 (31.3–47.4)	0.000*

Data presentation and abbreviation's spell are same as the description for Table 1.

* , *P*<0.05 vs pretreatment.

## Discussion

### Serum Mg, pre-diabetes, and diabetes

Correlation of reduced serum Mg level with diabetes have been extensively reported [20], [21], but there were also a few studies that did not show such correlation [14], [15]. By directly measuring serum and urinary Mg here we demonstrated the significantly low serum Mg level not only in T2D, but also in IFG, IGT, and T1D. Our study is inconsistent with a recent study [22]. They recruited 1453 adults from rural Victoria, Australia and measured their serum Mg with a level <0.70 mmol/L as hypomagnesaemia. After adjusting for confounders, they found that compared with control, hypomagnesaemia was 10.51-fold more common with new diabetes and 8.63-fold more common with known diabetes, but was not seen with IGT or IFG. They concluded that diabetes is associated with hypomagnesaemia, but not its pre-cursor states.

The inconsistence of our results with the above previous study [22] may suggest the different profiles among populations who from different countries (geographical difference), which may be related to habits of food intake in different populations. For instance, an early study from China [23] has indicated that patients with hypertension, IFG or diabetes took less Mg than controls and women also took less Mg than men. A recent meta-analysis of 13 prospective cohort studies involving 536,318 participants and 24,516 cases has clearly indicated the inverse association between Mg intake and T2D risk [17].

In the present study, we demonstrated for the first time that T1D patients also exhibited a significant low of serum Mg level compared to control. In terms of serum Mg levels in T1D patients there were only few reports. An early study measured the plasma concentration of copper, zinc and Mg in 37 patients with T1D and 25 healthy subjects and showed that mean plasma concentrations of copper and Mg were comparable between diabetic patients and controls [24]. This finding was supported by a recent cross-sectional study, showing that among the 50 T1D patients and 50 age-matched healthy children from Rafsanjan city, Iran, serum Mg level were more than 2 mg/dl with no significant difference between control and T1D patients [25]. However, a few other studies showed a significantly low serum Mg in T1D patients compared to control [26], [27]. The discrepancy may also be derived from populations since the first two were from Indian, and Iran, respectively, while the latter two from Slovak Republic and Poland, respectively.

In the present study, we have systemically compared the urinary Mg among subgroups, from which, we demonstrated the increased secretion of urinary Mg only in T2D and T1D patients compared to control.

### Serum or urinary Mg and diabetic complications

Several studies have indicated the positive correlation of low serum Mg levels with the incidence of various diabetic complications [13], [20], [28], [29]; therefore, we have compared the serum and urinary levels of Mg among subgroups of T2D patients without complication and with DN, DR or DPN. We did not find any significant difference for either serum or urinary Mg between uncomplicated T2D patients and T2D patients with either DN and DR or DPN. An early study also reported that there was no significant difference for serum Mg among three groups [10]: uncomplicated T2D (*n* = 30, serum Mg: 1.67±0.084), T2D with coronary atherosclerosis (*n* = 30, 1.60±0.072), and with peripheral vascular disease (*n* = 30, 1.71±0.085). However, there were also reports that showed the low level of serum Mg in T2D with food ulcer [20] and retinopathy [30] compared to uncomplicated T2D patients. For the discrepancy among these studies, the following reasons may be considered:

The population from different countries may be the first major factor. For instance, Sharma et al. have demonstrated that in 50 T1D and T2D patients with or without complications and 40 normal healthy persons, serum Mg was significantly low in diabetes with complication than without complications [30], which is support of a few previous studies [31], [32]. However, there was also a study that showed that low serum Mg was not associated with the occurrence of retinopathy in black Africans [14]. Second the duration of diabetes may affect such association. For instance, Sharma et al have demonstrated the during of diabetes with serum Mg were inversely related [30]. In addition, mean serum Mg level was found to be lower among those with known diabetes than those with new diabetes, which also suggests the length of diabetes may affect the serum Mg [10].

### Association of Mg with Ca in either serum or urine

In the present study, we demonstrated the significantly positive association of serum Mg with serum Ca in T2D patients, and of urinary Mg with urinary Ca in controls and those with either IGT or T2D. A positive correlation of urinary Mg with urinary Ca has been reported in an early study [33]. In a group of Nigerian diabetic patients, urinary excretion fractions of Mg and Ca were significantly increased in the diabetic group compared with controls with a significant positive correlation between Mg and Ca. We also demonstrated the increase secretion of Mg in urine for both T1D and T2D patients (Fig. 1B) and a strongly positive correlation of urinary Mg and Ca (Fig. 3B). The results from our study and others suggest that the renal tubular reabsorption of Mg and Ca is reduced in diabetic patients resulting in increased urinary losses of the two divalent cations.

In animal model of streptozotocin-induced diabetic rats [34], the increased urinary secretion of Mg and Ca was mechanistically associated to the increased gene expression and protein abundance of Ca and Mg transporters in the kidney. Insulin administration completely corrected the hyperglycemia-associated hypercalciuria and hypermagnesiuria, and reversed the increase of Ca and Mg transporter abundance. Therefore, a recent analysis of the interaction between serum Mg and Ca, defined as Mg×Ca, revealed that serum Mg and Ca, and Mg×Ca all had significant negative correlations with eGFR with the Mg×Ca showing the strongest correlation with eGFR [35].

### Effect of statin treatment on serum or urinary Mg

There was an early study that showed a reduction trend of serum Mg in the T2D patients treated with 4-month simvastatin treatment (*n* = 24) compared to T2D patients treated with placebo [36]. In the present study, we found no reduction of serum Mg, but significant reduction of urinary Mg in the T2D patients treated with simvastatin. We also found the significant reduction of serum Ca, but not significant reduction of urinary Ca in the patients treated with simvastatin (Table 5).

The above findings suggest that there was a risk for reducing either serum Mg or urinary Mg. Since we have appreciated, based on the above discussion, that Mg appears to play a vital function in the prevention of insulin resistance, diabetes and diabetic complications; In addition, Mg has been also reported to have anti-inflammatory and statin-like effect as well as the stimulating effect of Mg at physiological level on the statin passive diffusion into hepatocytes and their pharmacological actions on cholesterol biosynthesis [37]. Therefore, combination of statin administration with supplementation of certain amount of Mg may be required to avoid the reduction of the Mg level either in the blood or urine caused by supplementation with statin alone. In fact, the combination of Mg with a statin has been recently suggested as a potential and seemingly-promising avenue to reduce cholesterol, C-reactive protein, and cardiovascular disorders [38].

## Conclusions

The effect of Mg deficiency on the prevalence of metabolic syndrome and diabetes as well as diabetic complications has received a great attention. Mg as the fourth most abundant cation in the body plays a fundamental role as a cofactor in various enzymatic reactions involving energy metabolism, including insulin secretion, binding, and activity. Chronic Mg deficiency has been associated with the development of insulin resistance. The present study investigated, for the first time, the serum and urinary levels of Mg in different groups of the Chinese population who live in the Northeast areas of China. We found that: (1) serum Mg levels in the population with IGT, IFG, T2D, and T1D are significantly lower than that of healthy control. The increased secretion of urinary Mg was only found in T2D and T1D patients as compared to control; (2) In terms of diabetic complications, decreased serum Mg level and increased urinary Mg level in T2D patients was independent of the existence of either DN, DR, or DPN; (3) Serum Mg levels are strongly correlated with serum Ca levels only in T2D patients no matter with or without existence of DN, DR or DPN; Urinary Mg levels are strongly associated with urinary Ca levels in control, IGT patients, and T2D patients with and without existence of DN, DR, or DPN; Simvastatin treatment reduced serum Ca levels and urinary Mg levels in T2D patients.

Therefore, the potential impact of Mg in metabolic syndrome, diabetes and diabetes-related or no-related cardiovascular disorders needs to be received special attention. The abundance and low cost of the supplement, the relatively low side effect profile and the paucity of information in the literature about this common mineral suggest that more studies should be conducted to determine its safety and efficacy. Furthermore, the optimal combination of Mg supplementation with other efficient medication need to be developed to efficiently prevent and treat diabetes and diabetic complications.
